# Application of Fluorescence Lifetime Imaging Microscopy of DNA Binding Dyes to Assess Radiation-Induced Chromatin Compaction Changes

**DOI:** 10.3390/ijms19082399

**Published:** 2018-08-14

**Authors:** Elham Abdollahi, Gisela Taucher-Scholz, Burkhard Jakob

**Affiliations:** 1 Department of Biophysics, GSI Helmholzzentrum für Schwerionenforschung GmbH, 64291 Darmstadt, Germany; E.abdollahimirzanagh@gsi.de (E.A.); g.taucher-scholz@gsi.de (G.T.-S.); 2 Department of Biology, Technical University of Darmstadt, 64287 Darmstadt, Germany

**Keywords:** FLIM microcopy, Hoechst 34580, Syto 13, chromatin compaction, histone deacetylation inhibitor (HDACi), irradiation, pile-up

## Abstract

In recent years several approaches have been developed to address the chromatin status and its changes in eukaryotic cells under different conditions—but only few are applicable in living cells. Fluorescence lifetime imaging microscopy (FLIM) is a functional tool that can be used for the inspection of the molecular environment of fluorophores in living cells. Here, we present the use of single organic minor groove DNA binder dyes in FLIM for measuring chromatin changes following modulation of chromatin structure in living cells. Treatment with histone deacetylase inhibitors led to an increased fluorescence lifetime indicating global chromatin decompaction, whereas hyperosmolarity decreased the lifetime of the used dyes, thus reflecting the expected compaction. In addition, we demonstrate that time domain FLIM data based on single photon counting should be optimized using pile-up and counting loss correction, which affect the readout even at moderate average detector count rates in inhomogeneous samples. Using these corrections and utilizing Hoechst 34580 as chromatin compaction probe, we measured a pan nuclear increase in the lifetime following irradiation with X-rays in living NIH/3T3 cells thus providing a method to measure radiation-induced chromatin decompaction.

## 1. Introduction

Research in the past decade has revealed that in eukaryotes chromatin can be classified into higher-order structures with different compaction levels that are critical for the regulation of genome functions [1,2]. However, chromatin structure is not static, but subjected to changes in response to varying environmental conditions. In this respect, chromatin imposes profound impacts not only on transcription and replication, but also on the DNA damage response [3]. Chromatin compaction governs the accessibility of DNA break sites and it was even proposed that densely packed heterochromatin might be refractory to repair [4,5].

DNA double strand breaks (DSBs) as induced by ionizing radiation are considered to be one of the most severe forms of DNA damage. Even more critical are DSBs induced by high linear energy transfer (LET) radiation like alpha particles or heavy ions, as this type of radiation gives rise to more clustered DNA damage compared to low LET irradiation [6,7,8,9]. An improper repair of radiation-induced DNA-DSBs can jeopardize the integrity and stability of the genome and may ultimately lead to malignant transformation [10,11]. The understanding of molecular mechanisms and regulation of DNA damage repair within the chromatin context is one of the most important topics in radiation biology.

The application of chromatin compaction assays in living cells might help to address and elucidate the role of chromatin structure and its dynamics in the regulation of DNA repair. Live cell microscopy combined with online irradiation constitutes a functional approach to inspect fast responses to DNA damage and repair dynamics, because it can be performed immediately and without laborious repositioning [12,13,14]. A number of recent studies have applied imaging of fixed or living samples to address radiation-induced decompaction taking advantage of highly condensed heterochromatin which becomes clearly visible in murine or fruit fly cells upon DNA staining or tagging of heterochromatic protein 1 (HP1) [15,16,17]. A radiation-induced heterochromatic decondensation was described in line with the relocalization of damage to the heterochromatin/euchromatin interface for repair [15,16]. In contrast to the robust observation of a local chromatin decompaction deduced from a depletion of heterochromatic DNA staining after charged particle irradiation, rather moderate irradiation-induced changes in chromatin decondensation are measured by intensity-based methods in living cells [15,17]. A quantitative analysis is further hampered due to staining variability and the intrinsic problem of discriminating between actual chromatin decondensation and a reduction of available dye binding sites. Thus, a more robust readout independent of the intensity information and generally applicable in living cells without the necessity of genetic modifications is highly desirable.

There are several factors challenging the capture of high quality fluorescence images and the quantification of the obtained intensity information. These include fluctuations in the light source or in the fluorophore concentration within the sample, autofluorescence, fluorophore photobleaching, limitation in detector sensitivity, and the conditions for sample preparation [18]. Fluorescence lifetime imaging microscopy (FLIM) is a technique that can address some limitations of fluorescence microscopy related to fluorophore intensity fluctuation and autofluorescence background [19]. In FLIM, the time the electron spends in its excited state in the chromophore, called “fluorescence lifetime” is recorded in addition to the fluorescence intensity [20]. Therefore, FLIM is considered to be less influenced by artifacts arising from scattered light, photobleaching, nonuniform illumination or variations in excitation intensity, as these parameters influence the intensity, but leave the fluorescence lifetime of the chromophore largely unaffected [20]. Technically, FLIM can be measured either in the time domain or in the frequency domain. Whereas in the frequency domain a phase shift between synchrony of the modulation of the excitation source and the detector is employed, in the time domain either a gated integration technique or time correlated single photon counting (TCSPC) are used to measure the intensity decay. In this case most of the conventional instrumentation used for steady state fluorescence microscopy is not appropriate, since FLIM detects the lifetime of a fluorochrome in the range of few nanoseconds [21]. The fluorophore’s lifetime is an intrinsic property of the molecule, but it can be influenced by external factors that modify its emission rate constant. These include changes in the microenvironment surrounding the fluorophore, like pH, viscosity, changes in molecular crowding, or, most frequently used, the close vicinity of a second chromophore accepting the excitation energy by Förster resonance energy transfer (FRET) all of which can hence be studied using lifetime measurements. The increasing amount of interest in this powerful technique is documented in the review paper of Borst [22], where various FLIM applications are described. Regarding the chromatin compaction, several approaches have been described for the use of FLIM or FLIM-FRET techniques to read out the chromatin status in cells under different conditions or treatments [23,24,25,26,27,28].

Recently, we established a FLIM-based chromatin compaction assay using DNA binding dyes by which we successfully demonstrated that heterochromatin gets locally decondensed at the sites of ion traversal [29]. This finding was indicated by an increase in the lifetime data in nuclei fixed following irradiation. In this study, using this FLIM-based chromatin compaction assay, we quantify the dynamic ranges of promising single organic DNA dyes such as Hoechst 34580 upon modification of the chromatin compaction status in living cells. Furthermore, we show here that especially in inhomogeneous samples even moderate global count rates can influence the measured lifetimes, alike the impairment of TCSPC-FLIM data due to counting loss and pile-up effects previously reported at higher count rates [20,30]. Therefore, we applied a relative simple mathematical correction on a pixel to pixel basis for the aforementioned effects in order to get more reliable FLIM readouts. Using this approach, a global chromatin decompaction is shown in living NIH/3T3 cells following X-ray irradiation.

## 2. Results

### 2.1. Sensitivity of the Organic Dye Sensors upon Chromatin Modulation

To measure chromatin compaction states in living cells, Förster resonance energy transfer (FRET) between histones differently tagged with protein chromophores has been mainly used [23,24,28,31]. Here, we provide a FLIM protocol that is more universal by using cell permeable dyes e.g., Hoechst 34580, which bind to the minor groove of DNA, thus avoiding transient transfection or the generation of stable cell lines for sensor expression. To benchmark and compare the dynamic range of different cell permeable organic dyes as chromatin compaction sensors, we took advantage of the modulation of chromatin known to be induced by histone acetylation or changes in cation concentration (osmolaric changes). Valproic acid (VPA) is one of the most effective histone deacetylase inhibitors (HDACi) and its application induces chromatin decompaction mediated by increased acetylation of histones H3 and H4 at several residues [32].

**Figure 1:**
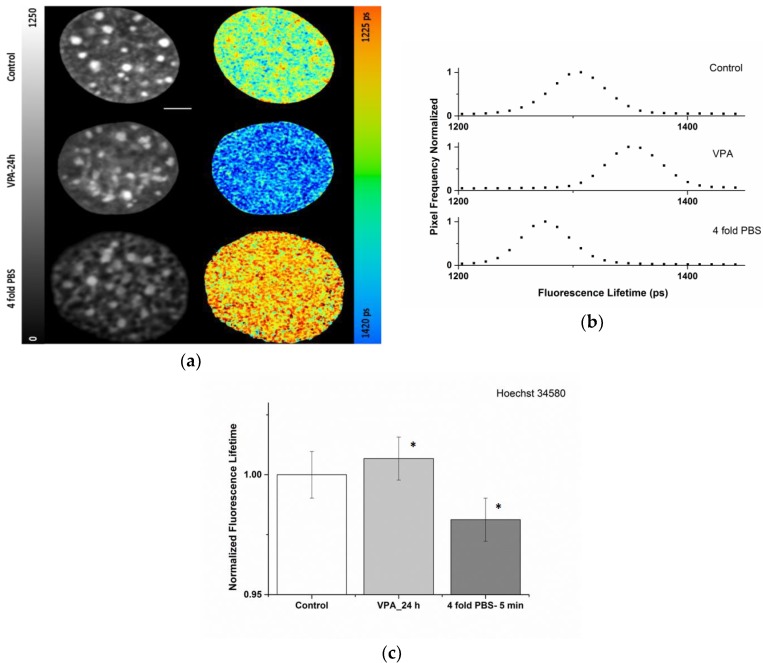
Modulation of the chromatin density of living NIH/3T3 cells stained with Hoechst 34580 and evaluation by fluorescence lifetime imaging microscopy (FLIM): (**a**) Intensity (left) and color coded lifetime images (scale ranging from 1225 to 1420 ps) of controls (upper row), upon treatment with histone deacetylase inhibitor, VPA, for 24 h (middle), or 4-fold Phosphate Buffer Saline (PBS, bottom). For 4-fold PBS chromatin compaction becomes obvious also in the intensity image showing the formation of condensed structures. Lookup table (LUT) at left side indicates corrected photon counts (0–1250). The fluorescence lifetime is shown in a continuous pseudo-color scale (right) ranging from 1225 to 1420 ps (**b**) Normalized frequencies of lifetime distributions from the nuclei of (**a**). Scale bar, 5 µm. (**c**) Quantification of relative Hoechst 34580 lifetime changes observed after histone deacetylase inhibitor (VPA) or hyperosmolarity (4-fold PBS). *n* = 20 for each condition. The lifetime values were normalized to control values. Asterisk (*) shows *p* < 0.05 (using Student’s *t*-test) compared to control. Error bars indicate mean ± SD.

Figure 1a,b shows FLIM images and fluorescence lifetime distribution (1372 ± 24 ps) of a nucleus of a living NIH/3T3 cell stained with Hoechst 34580 after VPA treatment. As a control, the fluorescence lifetime in normal medium without treatment was measured to be much lower (here 1322 ± 21 ps). Of note, control cells showed a clearly reduced lifetime in the chromocenters (1304 ± 15 ps for this nucleus), which represent compacted heterochromatin consisting of pericentric repeats, thus already indicating a compaction-dependent lifetime response. For comparison, a more general chromatin condensation was induced by exposing NIH/3T3 cells to hypertonic medium (~1280 mOsm/L; 1287 ± 24 ps). The typical shift in the lifetime values of the Hoechst 34580 upon the indicated treatments is apparent both in the color coded lifetime images (Figure 1a) as well as in the normalized frequency distributions of lifetime (Figure 1b) from the corresponding nucleus. In addition, the difference in fluorescence lifetime originating from heterochromatin versus bulk chromatin, clearly visible in the controls, was largely lost upon treatments.

The quantification and direct comparison of the relative and absolute Hoechst 34580 lifetime changes upon VPA or hypertonic treatment in a cell population is summarized in Figure 1c and Table 1. The results revealed that following VPA addition, the rise in the measured mean fluorescence lifetimes of Hoechst 34580 was in the order of 1%. Upon hyperosmolar chromatin compaction a general drop in the lifetime of Hoechst 34580 of around 2% was measured. The observed changes provide evidence that the lifetime of Hoechst 34580 is sensitive to modification of the chromatin status and support its usage as a chromatin compaction probe.

Not all tested DNA dyes did reflect chromatin compaction in their lifetime (See Table S1, list of tested DNA dyes). However, similar results to Hoechst 34580 could be obtained using Syto13. The fluorescence lifetime of Syto 13 responds to VPA or 4-fold PBS treatment in a range comparable to Hoechst 34580 (Figure S1). Syto 13 has the advantages that it can be excited by a 488 nm laser avoiding UV-irradiation and the fluorescence decay of this dye is largely mono-exponential, but it shows a clear contribution of RNA staining (e.g., in nucleoli). However this can be easily discriminated by its substantial longer lifetime (Figure S1) showing again the potential of the lifetime measurement.

### 2.2. Impact of Pile-up Effect and Counting Loss on Fluorescence Lifetime Readout

One of the major advantages of FLIM is its proposed independency on the local chromophore concentration in a given state. Accordingly, the staining variability at similar environmental conditions or a modulation of the excitation intensity should not affect the lifetime readout. A careful analysis revealed that in our measurements the fluorescence lifetime correlated with the applied laser intensity and thus the recorded photon numbers. For the lower laser power setting (2.7 µW) a general increase of the *t*_i_ values was observed compared to the higher laser power (10 µW) (See Figure S2 for the output power measurement of the 405 nm ps-diode laser). Especially at high counting rates (>10% of the laser pulse rate) this could be expected for start-stop or reverse start-stop TCSPC, as it is known that the first arriving photon is preferentially detected over a potential second photon in the same signal period [20,30]. This causes a so-called pile-up, yielding a distortion of the fluorescence decay curve resulting in an underestimation of the calculated lifetime [20,30]. We applied a simple mathematical correction according to the literature [20] where it is assumed that for small values of *P*, the recorded lifetime, τ_meani_, can be estimated by Equation (1) where *P* is the average number of photons per laser period and τ represents the correct fluorescence lifetime.

τ_meani_ ≈ τ (1−*P*/4) (1)

A more detailed discussion of the distortion effects of detector and electronic dead times as well as more sophisticated approaches for higher *P* values can be found in the literature [33].

In addition, the number of recorded photons is reduced by counting loss, even at relatively low average count rates around 1 MHz, due to the dead time of the detector/electronics which is several times larger than the time window defined by the pulsing frequency of the laser. This is of relevance specially in in homogeneously stained samples where local count rates may by far exceed the mean values. In these cases, counting loss leads to a reduction in image contrast. Even if counting loss is not directly contributing to a diminished lifetime, the non-detected photons must be taken into account in a pile-up correction for the calculation of the probability of recording more than one initial incoming photon per laser pulse. A more detailed description of counting loss and pile-up effect has been given in [20,34,35].

**Figure 2:**
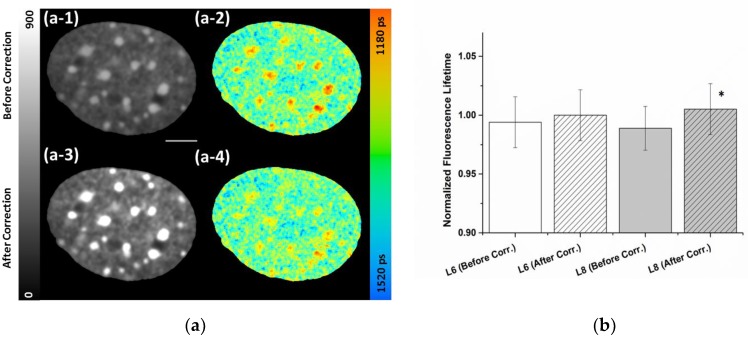
Influence of the pixel-wise correction of detector dead-time and pile-up. Confocal FLIM images of NIH/3T3 cell nucleus stained with Hoechst 34580 recorded at an average count rate of 0.9 MHz an 80 MHz laser repetition rate. (**a**) (a-1) uncorrected intensity image and (a-2) uncorrected lifetime image. (a-3) Intensity image with counting loss correction showing improved contrast and (a-4) lifetime image with the pile-up correction leading to increased values especially at high intensity areas. LUT at left side indicates photon counts for uncorrected (a-1) and corrected (a-3) intensities on same scale (0–900). The fluorescence lifetime is shown in a continuous pseudo-color scale (right) ranging from 1180 to 1520 ps. Scale bar, 5 µm. (**b**) Quantification of pile-up correction for different laser settings (L6: 2.7 or L8: 10 µW). Lifetime values were normalized to the corrected values of low laser intensities (2.7 µW). Asterisk represents (*) *p* < 0.05 (using Student’s *t*-test) compared to the uncorrected values of higher laser intensities. Error bars indicate mean ± SD; *n* = 15.

Higher local count rates compared to average originate mainly from bright areas (e.g., chromocenters) in the detection field, but also the dark areas outside the nuclei contribute to a reduction of the average count rates. To deal with this problem, we applied a mathematical correction for counting loss and pile-up taking the corrected number of photons at each pixel into account. Figure 2 illustrates the abovementioned effects and shows the results of the pixel-wise correction at an average uncorrected count rate of 0.9 MHz, thus far below the 10% of the 80 MHz laser pulsing. As seen in the panel (a-1) and (a-3), the correction of the counting loss clearly enhanced the contrast. Panel (a-2) shows the uncorrected lifetime image recorded at an average count rate of 2 MHz. The corresponding lifetime image with pile-up correction is depicted in panel (a-4). An average lifetime correction of about 0.1% was applied. However, at the high intensity chromocenters a lifetime correction value of around 2% became evident, indicated by a rise in the lifetime values (Figure 2, panel (a-2) and (a-4)). Using the correction, the compaction-dependent lifetime differences observed at chromocenters were attenuated but still clearly visible, proving that despite pile-up slightly affecting the calculated absolute values of the lifetime, the observed chromatin compaction-dependent lifetime was not artificially introduced by pile-up. Table 2 lists the measured photon numbers and lifetimes of the NIH/3T3 nucleus depicted in Figure 2 as well as bright areas (chromocenters) both before and after applying the counting loss and pile-up correction. As pile-up correction turned out to be important even at moderate count rate, this correction was applied to all measurements and images throughout this study, if not stated otherwise.

To examine the effect of the pile-up correction in TCSPC-FLIM measurements in a more systematic manner, we performed FLIM measurements of the same nuclei at different laser settings/intensities (L6: 2.7 µW and L8: 10 µW; see Figure S2). Figure 2b shows the lifetime values normalized to the corrected values at the low laser setting. At the low laser power, only a minor pile-up effect is induced, reflected in the 0.6% lifetime change from 1337 ± 29 to 1345 ± 29 ps. However, the impact of the correction was significant (average of 1.7%) at the higher laser intensity (from 1330 ± 25 to 1352 ± 29 ps). Remarkably, even at 10 µW laser power a slight decrease of the mean fluorescence lifetime compared to the 2.7 µW already had become obvious in the uncorrected values.

### 2.3. Radiation-Induced Global Chromatin Decompaction Imaged by FLIM in Living Cells after X-ray Irradiation

In order to study the effect of sparsely ionizing irradiation on chromatin structure in living cells, the FLIM setup was integrated in a cabinet equipped with a 35 kV X-ray tube. To obtain better statistics, several nuclei of NIH/3T3 cells were randomly selected, pre-irradiation images were acquired, and the coordinates saved in each dish before irradiation. Cells were then irradiated at high dose rate (35 kV, 10 Gy, ~1 Gy/s), repositioned and a second lifetime image of the same nuclei was acquired. The recordings were done in the time window 1–20 min following irradiation. As a prerequisite for monitoring small radiation-induced changes, the impact of the repetitive FLIM measurements on the measured fluorescence lifetime under the applied conditions was inspected. To do so, randomly selected and recorded nuclei were mock irradiated, revisited, and imaged for a second time to detect changes on the readout introduced by the first illumination and imaging procedure. The results validated that a second scanning had only minor impact (<0.5%) on the lifetime of Hoechst 34580 (See Figure S3).

**Figure 3:**
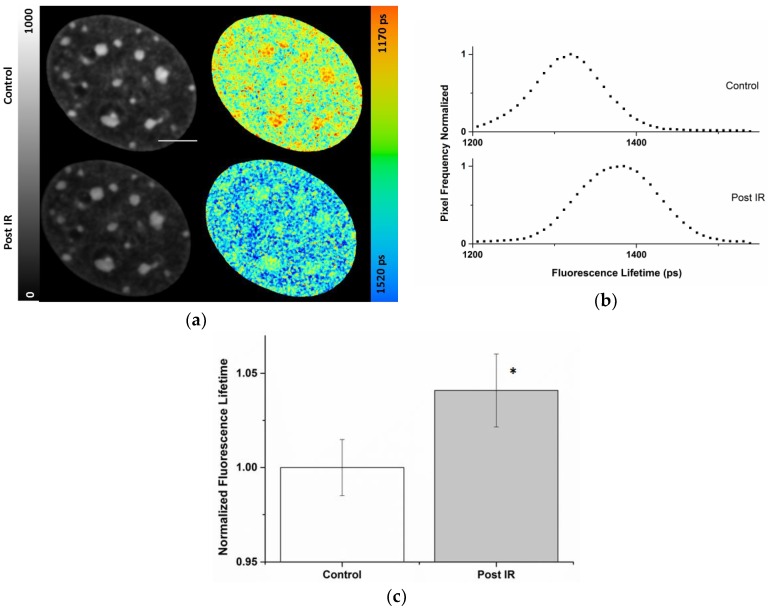
Increased Hoechst 34580 lifetime indicating a global chromatin decompaction after irradiation with X-rays. (**a**) Intensity and lifetime images of the same living NIH/3T3 nucleus before and after 10 Gy X-rays irradiation. Lookup table (LUT) at left side indicates corrected photon counts (0–1000). The fluorescence lifetime is decoded on a continuous pseudo-color scale ranging from 1170 to 1520 ps (right). (**b**) Normalized frequencies of lifetime values from the nuclei of (**a**). Quantification of the lifetime indicated a global shift in the lifetime distribution of the Hoechst 34580 after irradiation (**c**). Mean relative increase of fluorescence lifetime of Hoechst 34580 in NIH/3T3 nuclei upon irradiation with 10 Gy X-rays. Asterisk shows (*) *p* < 0.05 (using Student’s *t*-test) compared to pre-irradiated nuclei. Scale bar, 5 µm. Error bars indicate mean ± SD; *n* = 20.

Figure 3 shows an example of an NIH/3T3 nucleus prior to and six minutes post 10 Gy X-rays. The fluorescence lifetime images of Hoechst 34580 (indicated by color coding in Figure 3) showed a shift from 1331 ± 39 to 1390 ± 46 ps after 10 Gy X-rays. Interestingly, this increase affected the whole nucleus globally and did not arise from locally confined changes. This finding was also validated by the general shift of the nuclear lifetime distribution to higher values shown in Figure 3b. The nuclear wide average fluorescence lifetimes of Hoechst 34580 revealed a significant increase (*p* < 0.05) from 1344 ± 20 to 1399 ± 26 ps (Figure 3c) upon irradiation, indicating a generally induced global chromatin relaxation. The mean relative global increase (~4%) in Hoechst 34580 lifetime upon irradiation is shown in Figure 3c. In the intensity images no significant changes in the chromatin structure became evident upon irradiation (see also Supplemental Figure S8), showing the superior sensitivity of the lifetime information.

## 3. Discussion

In the nuclei of mammalian cells, many biological processes like gene expression or transcription, senescence, differentiation, and even cancer development are regulated by chromatin structure [2,10,36]. Due to this widespread impact in biology and particularly in many human diseases, improving the methods to study chromatin compaction is highly relevant. So far, various approaches have been utilized to address chromatin organization, e.g., nuclease digestion assays [37,38], DNA denaturation dependent staining using Acridine Orange in fixed cells [39], different forms of electron or super-resolution microscopy utilizing DNA dyes or antibodies [40,41,42,43,44], or fluorescence intensity based measurements [45]. Recently, also FLIM has been applied to get insights into the chromatin structure and its reaction upon different stimuli [23,24,25,26,27,28,29,31].

Several studies have suggested a protective effect of compacted chromatin on the generation of double-strand breaks by radiation [46,47,48,49]. Besides its influence on damage induction, chromatin organization was shown to play a role in damage signaling and repair of the induced DSBs, both in respect to the location as well as utilized repair pathways [15,16]. In this context, experimental evidence on radiation-induced chromatin decondensation has been provided using different techniques [16,29,50,51].

In our previous studies, we reported that shorter lifetime of some organic fluorescent DNA binding dyes, e.g., Hoechst 34580, are associated with heterochromatic chromocenters in mouse cells [29], which is also shown here for Hoechst 34580 (Figure 1, Figure 2 and Figure 3) as well as Syto 13 (Figure S1). This is in line with recent studies which described the application of organic DNA dyes as FLIM sensors for chromatin structure [26,27]. However, the molecular mechanism behind the lifetime-dependency of the single dyes on chromatin compaction is not known. It might originate from molecular collisions based on local crowding effects thus leading to nonradiative quenching [52]. Presently, we cannot exclude a potential influence of other environmental factors affecting the stability of the excited states. These factors might act differentially on various dyes. As by binding to DNA the dyes are in general conformationally stabilized giving rise to the enhanced fluorescence upon binding, a release of the dye during treatment might lead to a diminished lifetime. For Hoechst 34580 and Syto13 we could show that free, unbound dye present in the incubation medium (1 µM) has no major impact on the fluorescence lifetime readout (Figure S4), indicating that the unbound fraction is very efficiently quenched and not contributing to lifetime changes due to its very low fluorescence intensity.

Further issues might affect the fluorescence lifetime. For example, changes in ionic strength during treatment or local pH changes induced by the illumination of the chromophores during experiments [53] could potentially be responsible for the observed differences. Changes in pH have been demonstrated to influence spectral characteristics by protonation of DNA dyes [53] or by inducing dynamic melting of the double stranded DNA due to a temporal disruption of H-bonds leading to a dynamic dye binding behavior [53]. In both cases, massive pH changes (below pH 4) were applied.

By using lifetime measurements in fixed cells to decouple chromatin compaction changes from the potential environmental influence, we could show that for Hoechst 34580 a moderate variation of the pH value (from pH 6.4 to 8.5) did not induce larger variations in the lifetime readout (Figure S5). Furthermore, such large pH changes as necessary for the effects described above would rather not be expected in the variation of the local chromatin environment during normal measurements. In addition, we also verified that Hoechst 34580 did not show a significant lifetime modification upon modulation of osmolarity/ionic strength by going from 0.5× PBS to 4× PBS (Figure S5), thus supporting the view that the observed reduction in lifetime upon hyperosmolarity in living cells (Figure 1) depends on the induced chromatin compaction. In contrast to Hoechst 34580, Syto13 revealed a certain dependency on this type of modulation in fixed samples (Figure S6).

In conclusion, at least for Hoechst 34580, which also shows superior binding stability compared to Syto 13 (not shown), the observed changes in fluorescence lifetime in the life cell experiments most likely reflect changes in chromatin compaction.

Regarding the limitations due to chromophore proximity aspects, we have to state, that not all structural dimensions of the chromatin can be probed by the FLIM technique. Here, as for FLIM-FRET, the observed modulation of the fluorescence lifetime most probably results from modulation in the near (nm) vicinity—so it is tempting to speculate that changes in the density of the recently described chromatin clutches [40,54] are responsible for the observed fluorescence lifetime changes. Rearrangements at larger scales will most probably not influence the fluorescence lifetime and need to be addressed using alternative approaches. We must bear in mind that although the chromophores are sensing changes on the nm scale, the maximal resolution of the FLIM method applied is diffraction limited as a point scanning confocal microscope is used. We used nearly optimal sampling according to the Nyquist criteria (42–62 nm) leading to an optical resolution of around 200 nm. However, as for all live cell experiments, the internal motion of the chromatin [13,55] during the recording time (here 40 s), and the routinely applied binning for the fitting of the decay curves to obtain better photon statistics will sacrifice some of the theoretical resolution. Nevertheless, localized submicron lifetime changes could be visualized after charged particle irradiation [29].

In TCSPC-FLIM measurements, at higher local fluorescence intensities the pile-up effect has an impact on the recorded lifetime values by preferentially recording the early photon in a laser pulse interval. Thus, care has to be taken not to introduce lifetime variations by different fluorescence intensities leading to elevated count rates at brighter spots. We demonstrate here that pile-up needs to be taken into account also at moderate average detector count rates (Figure 2) even if high excitation frequencies (80 MHz) are used, which are considered to minimize pile-up due to reducing the probability to have more than one photon per laser period. Especially for inhomogeneously stained samples, local detection rates can exceed the average count rate by far. In our example, the actual count rate at the chromocenters was 4.4 MHz compared to 0.9 MHz overall at 80 MHz pulsing. Calculated correction factors have been found to be in the same order of magnitude (~1–2%) at bright spots as commonly observed differences for different chromatin compaction (e.g., Figure 1). This is in agreement with the authors of a previous paper [33] who showed that slight changes in lifetime values of Atoo647nN labeled actin filaments after dead time correction even at count rates well below 10% of the laser repetition rate in a TCSPC system with constant dead time. Therefore, all recorded and shown images and reported values throughout the study show corrected values, if not stated otherwise. However, we could show that after applying the correction (assuming the conservative approach of taking the count loss corrected detection rate) a difference in Hoechst 34580 values was still evident at sites of compacted chromatin (Table 2), indicating that the observed reduction in the lifetime not artificially introduced. In addition, we could show that counting loss introduced by unavoidable detector dead time leads to a degradation of the image contrast and might prohibit a correct interpretation of intensity values. Applying the relatively simple mathematical correction on a pixel to pixel base incorporating the average frequency of photon detection in a given pixel during a laser period resulted in more reliable outcomes (Figure 2, Table 2) likely suitable for many biological applications. A detailed discussion of the origin and a more sophisticated mathematical approach for corrections has been described [34,35]. This would allow the reconstruction of the decay curves and lifetime components even at very high count rates, which were not encountered in our measurements. Reverse start-stop TCSPC sometimes shows inter-pulse pile-up due to variable electronic (TAC/ADC) dead times that are difficult to correct for [33]. However, at the high repetition rates and the moderate photon counting rates used during our experiments, no step-like distortions of the decay curves have been observed indicating no major contribution. Alternative instrumental approaches using independent timers for the synchronization and the detector pulses have recently been developed to avoid Pile-up effects [20,33], thus making the dead time corrections easier. Overall, especially when small differences in lifetime values can be expected, as shown here for instance for the internal nuclear variation or radiation-induced changes, the pile-up and counting loss corrections should be taken into account.

Several studies applied a FLIM-based assay and described a reduction in the lifetime of H2B-GFP in the presents of co-expressed H2B-mCherry due to the occurrence of FRET [23,31]. The major disadvantage impeding the general usage of FP-tagged histones is the necessity of either applying transient transfection to the cells or the usage of established genetically modified cell lines. In contrast, cell permeable DNA binding dyes easily can be used in all cell lines of interest and also under conditions not allowing transfection.

Modulation of the chromatin structure by changing histone acetylation or hyperosmolarity revealed significant changes in the fluorescence lifetime of Hoechst 34580 (Figure 1) or Syto 13 (Figure S1) in accordance with the expectation. VPA treatment of NIH/3T3 cells stained with Hoechst 34580 showed an increase of about 1% of the mean lifetime (Figure 1c), and are thus in a similar range as the observed changes for FP-tagged histones reported in the literature [23]. It was shown before that the hyperacetylation induced via VPA/TSA resulted in a chromatin relaxation [23,32,56]. Thus, it can be concluded that this global chromatin decondensation is reflected by the measured increase in lifetime. Nevertheless, presently we cannot rule out that in the case of HDACi (Figure 1, Figure S1) the induced epigenetic modification of the histones can in addition directly influences the lifetime of the used dyes.

On the other hand, hyperosmolarity of NIH/3T3 cells revealed an approximate 2% decrease in the mean lifetime value of Hoechst 34580. Such a significant decline of this value is in line with the expected chromatin compaction which became also evident in the intensity images by the formation of void areas and the formation of filamentous structures (Figure 1a). Applying an alternative evaluation method of the intensity images using texture analysis based on the grey level co-occurrence matrix (GLCM) [57] confirmed these visual changes in some of the homogeneity parameters (Figure S7). By varying the spatial distance in the GLCM the most prominent changes that could be observed are at a length scale of 120–800 nm fitting to the observed fiber formation upon 4 × PBS treatment. Hyperosmolarity has been shown to induce chromatin compaction in living HeLa H2B-GFP cells [45]. There, a relative 60% decrease of the volume of chromatin after application of 4-fold PBS was described, supporting the FLIM data of this work. Consistent with the unaltered visual appearance after VPA (Figure 1), as well as after X-ray irradiation (Figure 3), no significant changes in our texture analysis could be observed (Figures S7 and S8), indicating that the FLIM readout was superior in detecting subtle chromatin changes.

Recently, in cells fixed immediately after irradiation, we could demonstrate that some organic DNA binding dyes can be favorably utilized to monitor spatially-confined ion irradiation-induced chromatin decompaction [29]. At the position of the ion traversals a remarkable local enhancement in the lifetime of Hoechst 34580 could be detected [29]. This was in line with the earlier observation of an eye-catching depletion in the intensity of DAPI or Hoechst 33342 at chromocenters traversed by accelerated ions, which was attributed to a local densely-ionizing radiation-induced decompaction of heterochromatin [15,17]. However, in contrast to the FLIM data, the purely intensity-based analysis has the inherent problem of not being able to distinguish between the decompaction of chromatin and a reduction of available dye binding sites. To study the chromatin response upon sparsely ionizing radiation, the FLIM system was coupled here to an X-ray source. Interestingly, in contrast to the irradiation with charged particles [15,17] or laser micro-irradiation [58,59], we did not observe locally confined responses but a global change by around 4% in the lifetime values of Hoechst 34580 in postirradiation FLIM measurements after X-rays. Interestingly, this rise in the lifetime was not restricted to the highly condensed chromocenters. The increased lifetime provides evidence for the loosening up of chromatin upon irradiation in living cells. This finding is in agreement with a recent study using super resolution localization microscopy, in which changes of the conformation of the chromatin and a decompaction of labeled sites in vicinity of ALU cluster were shown upon irradiation [42]. Whereas super resolution methods or electron microscopy clearly have the advantage of superior resolution and probing chromatin changes at different length scales, they normally rely on fixed specimens or require specialized buffer conditions which are generally not compatible with live cell measurements. Our FLIM approach using fluorescent DNA binding dyes allows for repetitive measurements (e.g., Figure 3) to measure changes upon external stimuli like damage induction, even if care has to be taken—as for all live cell experiments—that the illumination is not influencing the process under investigation. Depending on the experiment, compromises regarding the temporal and spatial resolution as well as the number of repetitive measurements might be necessary if kinetics is followed. For the repetitive measurements in Hoechst 34580-stained nuclei shown here (Figure S3), the recorded lifetime in the second scan stayed largely unaltered under the applied conditions.

A radiation-induced chromatin decompaction is thought to be a necessary prerequisite for damage signaling and the progress of DNA repair. The molecular mechanisms behind this chromatin change, the involvement of different remodeler complexes, and their interplay within the DNA damage response are the matter of ongoing research. FLIM might allow addressing the spatiotemporal dynamics of chromatin density changes during irradiation and can help to pinpoint the biological mechanisms and consequences of radiation induced chromatin changes in future studies. Besides radiation related questions, evaluation of the chromatin status is important in many biological applications. Measurements might comprise e.g., the evaluation in different cell lines or during processes involving epigenetic changes and/or chromatin alterations like differentiation, carcinogenesis, or changes in the metabolic state. In this view, due to the plethora of biological responses and diseases influenced by chromatin organization, many fields might benefit from additional tools quantitatively addressing the chromatin compaction like the introduced lifetime measurements of organic DNA binding dyes.

## 4. Materials and Methods

### 4.1. Sample Preparation and DNA Staining

NIH/3T3 cells (ATCC, Manassas, VA, USA) were cultured in DMEM medium and supplemented with 10% fetal calf serum (FCS, Biochrom AG, Berlin, Germany), 4.5 g/L glucose, stable glutamine, and Na-pyruvate on Ø35 mm glass bottom petri dishes (Greiner bio-one, Frickenhausen, Germany). For FLIM measurements, 1.5 × 10^5^ or 0.7 × 10^5^ cells were seeded one or two day(s) prior to the experiments, respectively.

For live cells measurements, cells were stained directly before measurements with 1 µM Hoechst 34580 (Biomol GmbH, Hamburg, Germany) in cell medium for 1 h. After staining and incubation, the dye solution was removed and fresh medium was supplied before FLIM measurements were carried out.

In case of measurements in fixed samples, chemical cross-linking of cell was done after removal of medium and washing by Phosphate Buffer Saline (PBS) using 2% paraformaldehyde in PBS for 15 min.

#### 4.1.1. Histone Deacetylation Inhibitors, Valproic Acid

VPA (Sigma, Taufkirchen, Germany) was dissolved in PBS and diluted in Dulbecco’s Modified Eagle’s Medium (DMEM) including 2% FCS to a concentration of 1 mM. VPA was added to the cells for 24 h prior to the DNA staining. Cells were washed with fresh medium and DNA dye incubation started as described above.

#### 4.1.2. Hypertonic Treatment

Osmolaric changes were conducted after the DNA staining procedure. Directly at the FLIM microscope, 5 min prior to FLIM recording, cells were treated with 4-fold concentrated PBS.

### 4.2. Microscopy, Irradiation and Image Analysis

Fluorescence life time imaging of living cells was done using a DCS 120 scan head (Becker & Hickl, Berlin, Germany) as described previously [29]. A heated environmental chamber to control temperature (37 °C), humidity, and CO_2_ (5%) supply was used (Tokai Hit, Fujinomiya-shi, Shizuoka, Japan). Confocal images were recorded for 20–40 s in the FIFO mode using a 60× water immersion lens (NA = 1.2, Olympus, Tokyo, Japan) at a pixel size of 42–62 nm/pixel. Laser power was adjusted to give a mean photon count rate of the order of 10^5^–10^6^ photons/s. The FLIM setup is combined with a 35 kV X-ray tube (GE Inspection Technology, Ahrensburg, Germany) which was operated at 80 mA delivering a dose rate of about 1 Gy/s at the cell layer. Image analysis was done using SPCImage version 6.4 (Becker & Hickl, Berlin, Germany) and ImageJ software version 1.48v (Available online: http://imagej.nih.gov/ji). For Syto 13 a monoexponential and for Hoechst 34580 a bi-exponential fitting model at binning of 3 was used. Quality of fits was judged using Chi- square (χ^2^) test. Presented values are intensity weighted average lifetimes t_i_. To calculate mean lifetime and lifetime distributions the nuclei were manually segmented. For single nuclei, errors represent the standard deviation of the pixel values of the nucleus. For population values, error bars indicate the standard deviation of the mean values of the nuclei treated under identical conditions. Correction of pile-up and counting loss was done using Wolfram Mathematica version 9.0 for Microsoft windows (Available online: http://pacletserver.wolfram.com). Data were plotted using Origin pro software (Version 9.0.0 (32-bit) SR2 b87, Northampton, MA, USA) or Excel (Vers. 2010, Microsoft Corporation, Redmond, WA, USA). For texture analyses a home written macro based on the GLCM- Plugin (v.04; Julio E. Cabrera; Available online: https://imagej.nih.gov/ij/plugins/texture.html) was used. The length of the two orthogonal displacement vectors applied on a representative rectangular section of the intensity image of each nucleus were varied to cover different scales of textural changes.

## Figures and Tables

**Table 1:** Chromatin compaction modulation measured by FLIM.

Condition	*t*_i_ (ps)
Control	1330 ± 12
VPA-24 h	1342 ± 12
4-fold PBS	1308 ± 12

Mean fluorescence lifetime values *t*_i_ ± SD in nuclei stained with Hoechst 34580 shown in Figure 1c. Valproic acid (VPA).

**Table 2:** Pile-up and counting loss corrections of FLIM data.

Cell Compartment	*t*_i_ (ps) Pre-Correction	*t*_i_ (ps) Post-Correction	Photon Pre-Correction	Photon Post-Correction
Nucleus	1370 ± 26	1372 ± 24	293 ± 77	419 ± 186
Chromocenters	1306 ± 26	1340 ± 21	480 ± 68	885 ± 227

The recorded and corrected lifetime values and photons number of Hoechst 34580 in the nucleus and chromocenters (bright areas) depicted in Figure 2. Error represents mean ± SD.

## Supplementary Materials

Supplementary materials can be found at http://www.mdpi.com/1422-0067/19/8/2399/s1.

## Abbreviations

FLIM	Fluorescence Lifetime Imaging Microscopy
TCSPC	Time Correlated Single Photon Counting
FRET	Förster Resonance Energy Transfer
LET	Linear Energy Transfer
DSB	Double Strand Break
HDACi	Histone Deacetylase Inhibitor
VPA	Valproic Acid
PBS	Phosphate Buffer Saline
FP	Fluorescence Protein
LUT	Lookup Table
GLCM	Gray Level Co-occurrence Matrix
