# Supplementary material for: Application of Fluorescence Lifetime Imaging Microscopy of DNA Binding Dyes to Assess Radiation-Induced Chromatin Compaction Changes
Source: Int J Mol Sci. 2018 Aug 14;19(8):2399. doi: 10.3390/ijms19082399 (PMC6121443; doi:10.3390/ijms19082399)
Supplement: Supplementary file 1 [file ijms-19-02399-s001.pdf]

Table S1. List of DNA dyes used for DNA staining in living cells.

DNA Dye	Ex/Em	DNA Dye	Ex/Em
Hoechst 33342	350/461	Syto Orange 81	530/544
DAPI	358/461	Syto Orange 80	531/545
Hoechst 34580	392/440	Syto Orange 82	541/560
Nuclear Violet	401/459	Syto Orange 83	543/559
Syto Blue 41	430/454	Syto Orange 85	567/583
Syto Blue 42	433/460	Syto 64	599/619
BENA 435*	435/484	Syto 17	621/634
Syto Blue 45	455/484	Syto 59	622/645
Syto 13	488/491	Syto 61	628/645
Syto 16	488/518	Vybrant Ruby	638/686
Oxidize DHE	490/590	Draq 5	646/681
PicoGreen	502/523	Syto 62	652/676
Nuclear Green	506/534	SirHoechst	652/674
Cytrak Orange	520/615	Syto 63	657/673

* BENA 435 was kindly provided by Dr. Chi Hung Nguyen, UMR 176 CNRS-Institute Curie.

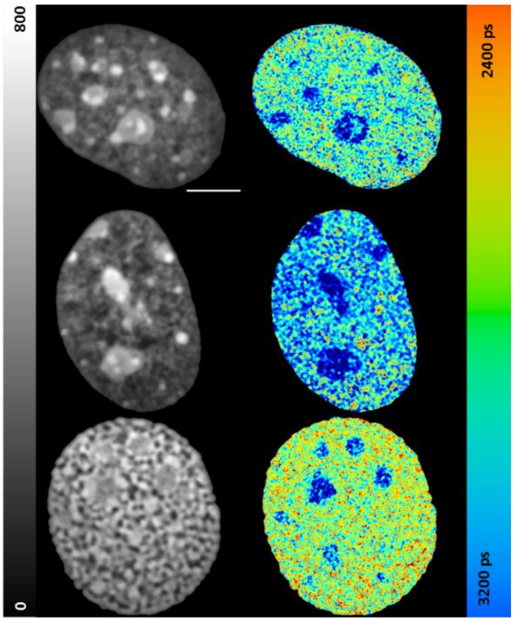

(**a**)

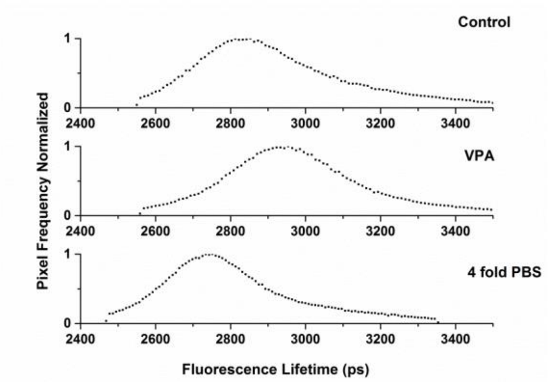

(**b**)

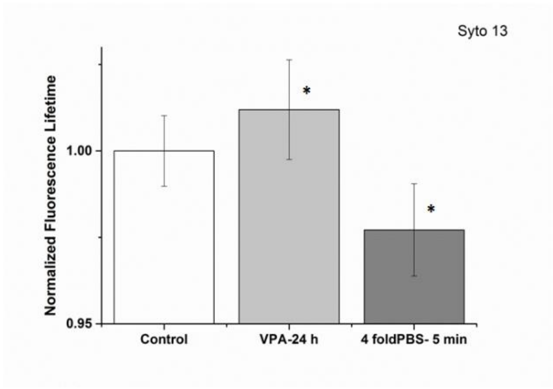

(**c**)

Figure S1. Modulation of the chromatin status of living NIH/3T3 cells stained with 1 µM Syto 13 incubated for 30 min and evaluation by FLIM: For Syto 13 lifetime data were fit by monoexponential decay curves. (**a**) Intensity (left) and color coded lifetime images upon treatment with histone deacetylase inhibitor VPA and 4 fold PBS. (**b**) Normalized frequency of lifetime distributions from the nuclei of (**a**). Scale bar = 5 µm. (**c**) Statistical significant changes of the lifetime of Syto 13 were observed after VPA and 4 fold PBS treatments. The lifetime values were normalized to the control value. Asterisks show $p < 0.05$ (using Student's *t*-test) compare with control. Error bars indicate mean ± SD.

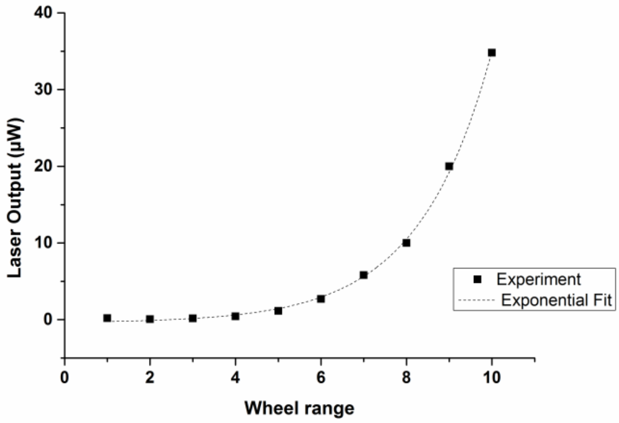

Figure S2. Output power of the 405 nm ps-diode laser measured after the 60×/1.2 NA water immersion objective lens at the imaging plane. Measurement was done using an Ophir Orion-PD powermeter (Ophir Spiricon Europe, Darmstadt, Germany) equipped with a PD-300-UV photodiode sensor. Increasing the ND-filter wheels from 1 to 10 showed an exponential rise in the output power of the laser in the range of 0.2 to 35 μW.

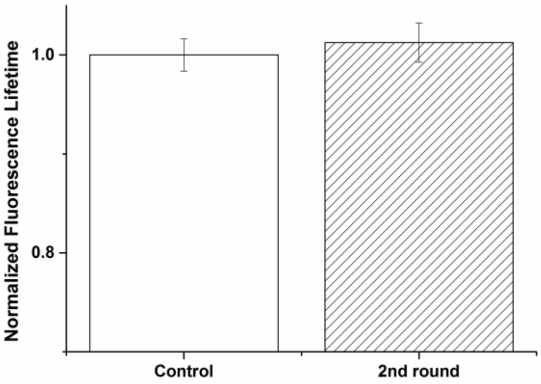

Figure S3. Successive FLIM measurements of un-irradiated living NIH/3T3 cells stained with Hoechst 34580. The fluorescence lifetime of Hoechst 34580 was normalized to the lifetime of the corresponding first measurement. Control showing largely unaltered Hoechst 34580 mean lifetime (n = 50). Error bars indicate mean ± SD.

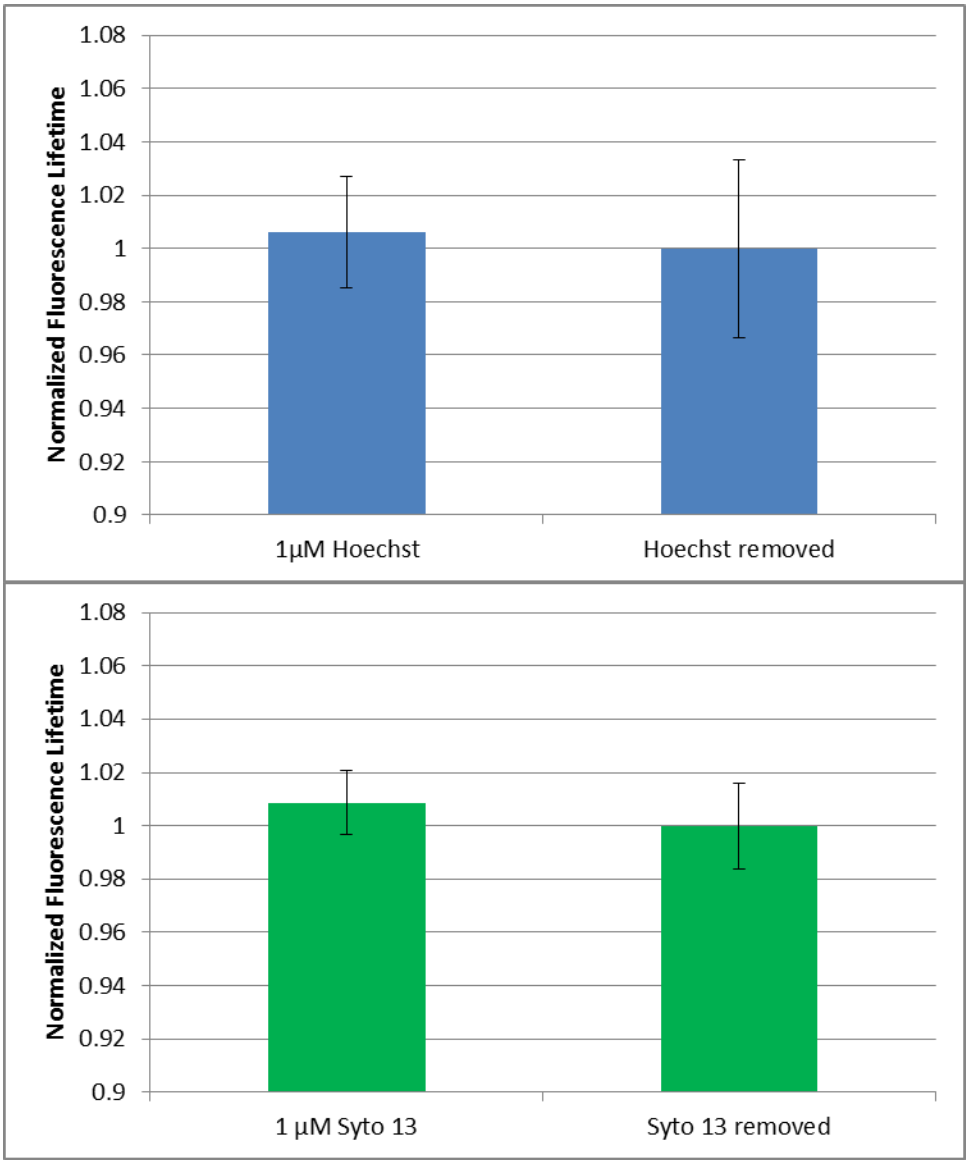

Figure S4. Influence of unbound dye on lifetime readout in U2OS cells stained with Hoechst 34580 or Syto 13. Lifetime was first measured after 1h incubation in 1 μM dye solution as described in the Methods section (unbound dye present, left column) and the staining solution was subsequently replaced with fresh PBS (dye removed). Second measurements (right column) were done up to 25 min after changing the conditions. The fluorescence lifetime was normalized to the lifetime after removal of dye. Measurements were obtained in the same dish; in the presence of 1 μM unbound dye, no reduction of the mean lifetime values was observed (n = 13–18). Error bars indicate mean ± SD.

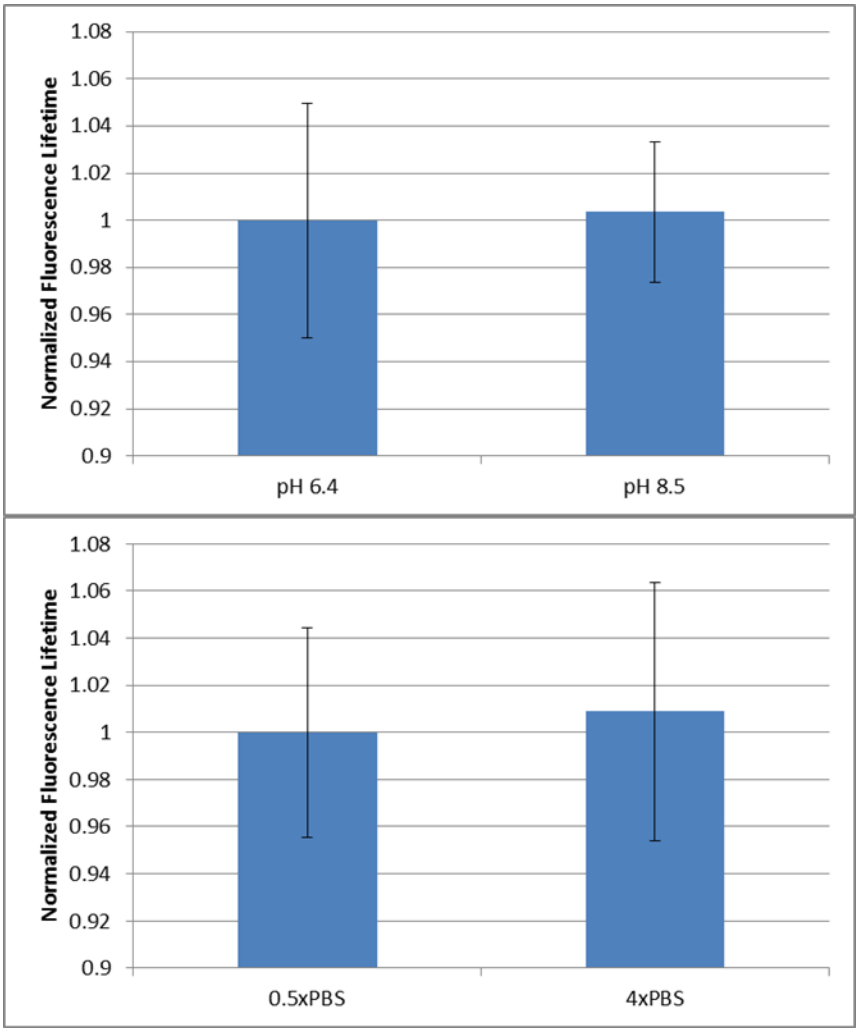

Figure S5. Influence of pH and ionic strength on lifetime readout in fixed U2OS cells stained with 1 μM Hoechst 34580. Cells were fixed and stained as described in Methods and the dye solution was then exchanged for fresh PBS adjusted to pH 6.4 (upper panel) or 0.5 × PBS (lower), respectively. The first measurement (left columns) was done up to 25 min after adjusting the conditions; second measurements (right columns) were performed up to 25 min after replacing the PBS solutions with fresh PBS pH 8.5 (upper panel)/or 4 × PBS (lower). Cells were washed with the corresponding buffer at each replacement step. The fluorescence lifetime was normalized to the lifetime of the first condition. Moderate pH changes as well as changes in salt concentration showed only minor influence on Hoechst 34580 lifetime values in fixed cells. (n = 14–20). Error bars indicate mean ± SD.

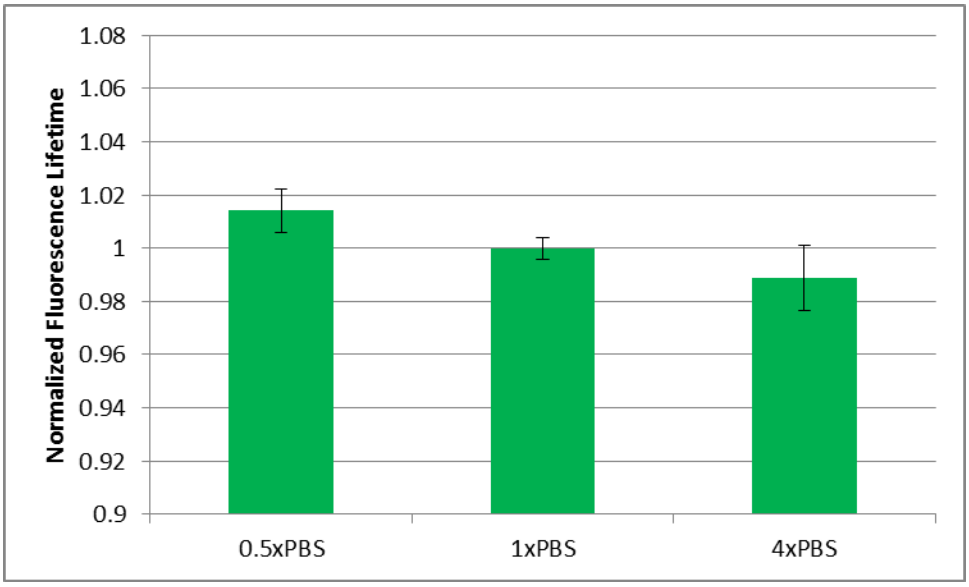

Figure S6. Influence of ionic strength on lifetime readout in fixed U2OS cells stained with 1 μM Syto 13. After staining fixed cells as described in Methods fresh PBS was added and a first measurement (middle column) was done up to 25 min thereafter. Second measurements (left columns) were performed up to 25 min after removal of PBS and subsequent addition of fresh 0.5 × PBS. Third measurements (right columns) were performed up to 25 min after replacement of the solution with fresh 4 × PBS. Cells were washed with the corresponding solution at each replacement step. The fluorescence lifetime was normalized to the lifetime at isotonic salt concentration (1 × PBS). The lifetime values of Syto 13 revealed a moderate decline with increasing ionic strength. (n = 15–16). Error bars indicate mean ± SD.

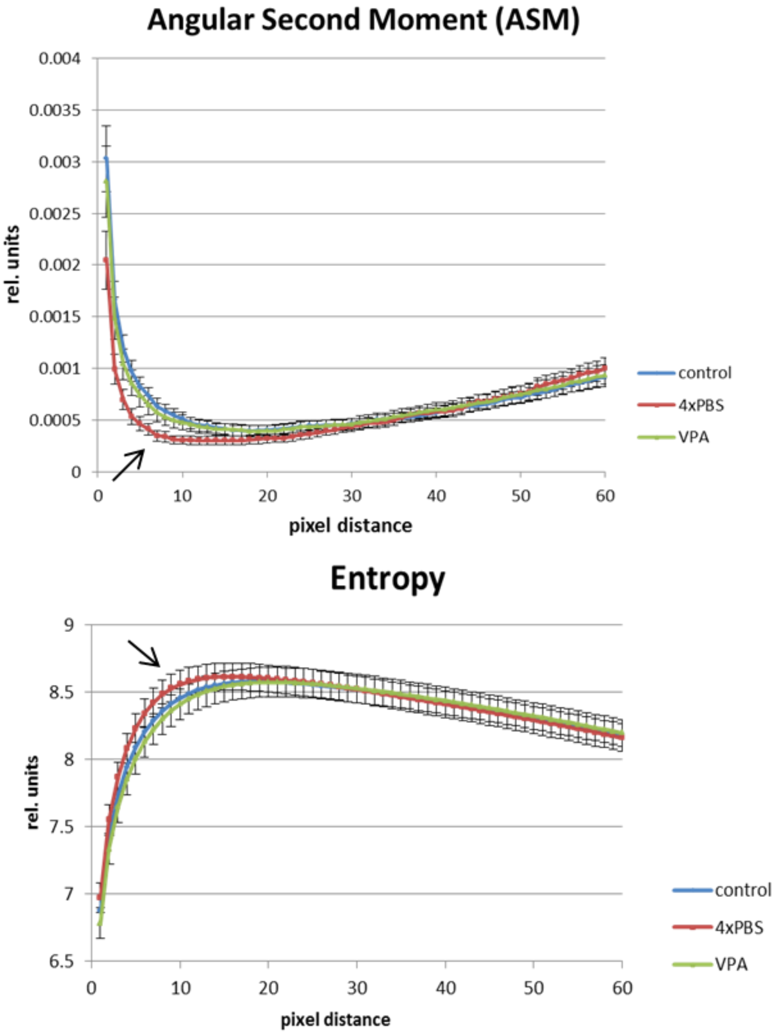

Figure S7. Texture analysis of intensity images of living, Hoechst 34580 stained NIH/3T3 nuclei. The homogeneity parameter Angular Second Moment (ASM) and the disorder parameter Entropy. By varying the pixel distance of the corresponding Gray level co-occurrence matrix (GLCM) small changes become evident especially in the ASM parameter after 4 × PBS in the range of 2 to 13 pixels (62 nm/pixel) corresponding to 120 to 800 nm (arrow). Control and VPA treated cell show nearly identical behavior of all tested length scales. Error bars indicate mean ± SE.

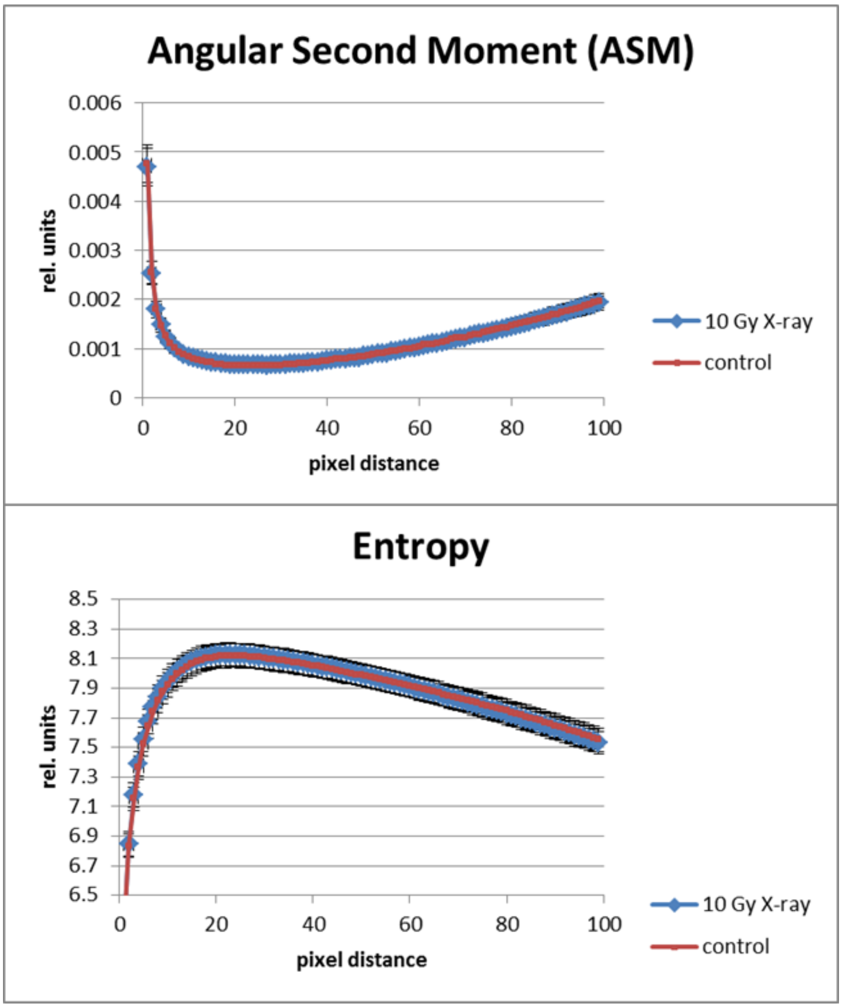

Figure S8. Texture analysis of intensity images of living, Hoechst34580 stained NIH/3T3 nuclei upon irradiation with 10 Gy X-rays. The homogeneity parameter Angular Second Moment (ASM) and the disorder parameter Entropy are shown for the same nuclei before and after irradiation. No significant changes in ASM or Entropy were induced by the irradiation, independent of the pixel distance (42 nm/pixel) of the corresponding gray level co-occurrence matrix (GLCM). Error bars indicate mean ± SE.
